# Sociodemographic, behavioural and health factors associated with changes in older adults’ TV viewing over 2 years

**DOI:** 10.1186/s12966-014-0102-3

**Published:** 2014-08-15

**Authors:** Benjamin Gardner, Steve Iliffe, Kenneth R Fox, Barbara J Jefferis, Mark Hamer

**Affiliations:** Health Behaviour Research Centre, Department of Epidemiology and Public Health, University College London, London, UK; Research Department of Primary Care & Population Health, University College London, London, UK; Centre for Exercise, Nutrition and Health Sciences, University of Bristol, Bristol, UK; Population Health Domain Physical Activity Research Group, University College London, London, UK; Department of Epidemiology and Public Health, University College London, London, UK

**Keywords:** Older adults, Sedentary behaviour, TV viewing, Screen time, Prospective

## Abstract

**Background:**

Of all age groups, older adults spend the most time watching TV, which is one of the most common sedentary behaviours. Such sedentary activity in older adulthood is thought to risk deterioration of physical and mental functioning, health and wellbeing. Identifying the characteristics of older adults whose TV viewing increases over time may help to target sedentary behaviour reduction interventions to those in most urgent need. Yet, studies of the factors associated with TV viewing have predominantly been cross-sectional. This study used a prospective design to describe changes in TV viewing over a two-year follow-up period, and to model socio-demographic, behavioural and health factors associated with observed changes in viewing time.

**Methods:**

A two-year follow-up of 6,090 male and female older adults (mean age 64.9 ± 8.9 years) was conducted in the English Longitudinal Study of Ageing, a cohort of community dwelling older adults. TV viewing time was self-reported at baseline and at follow-up. The sample was categorised according to baseline TV viewing duration (<2 hrs/d, 2 < 4 hrs/d, 4 < 6 hrs/d, ≥6 hrs/d), and the observed direction and extent of changes in viewing duration were described for each category. Socio-demographic, behavioural and health variables (socioeconomic status, depressive symptoms, disability, chronic illness, body mass index, physical activity, smoking), as measured at baseline, were entered into regression models as predictors of changes in TV viewing time between baseline and follow-up.

**Results:**

Mean self-reported TV viewing time increased from 5.32 ± 4.08 hrs/d at baseline to 5.53 ± 4.19 hrs/d at follow-up (p < 0.001). Forty-nine per cent of participants increased their TV viewing (23% of all participants by 60 minutes or more), 41% decreased their viewing, and 10% reported no change in viewing duration. Increases in TV viewing at follow-up were associated with lower socioeconomic status, presence of depressive symptoms, higher BMI, physical inactivity, and being a smoker at baseline.

**Conclusions:**

Findings call for the development of effective behaviour change interventions to counter increases in inactive TV viewing among older adults, and point to subgroups who may need to be prioritised for such interventions.

**Electronic supplementary material:**

The online version of this article (doi:10.1186/s12966-014-0102-3) contains supplementary material, which is available to authorized users.

## Background

Sedentary behaviour – i.e., low energy-expenditure activity undertaken in a sitting or reclining position [1] – is associated with adverse physical and mental health outcomes [2-4]. Even among the physically active, time spent sedentary is associated with higher waist circumference, blood pressure, and 2-hour plasma glucose [5,6]. Sedentary behaviour thus appears to have deleterious health effects even where physical activity recommendations are met [7,8], and so sitting time is now recognised as a health risk factor independent of physical activity [9]. Physical activity guidelines are increasingly incorporating recommendations to limit sedentary time [10].

Older adults are more likely than other age groups to be sedentary [11,12]. TV viewing, one of the most prevalent leisure-time sedentary activities [13], is particularly common in older adults. It is estimated that adults typically spend 60-70% of waking time in sedentary activities [14,15], and UK adults aged 65 or above spend an average 4 hours a day watching TV [16]. Given the sedentary nature of typical TV viewing episodes, studies have linked viewing time in older adulthood to poorer physical health, and greater depression, anxiety, and cognitive decline [17-21]. Substituting sedentary TV viewing time for non-sedentary activities has the potential to yield significant population-level health impacts among the elderly.

Cross-sectional comparisons across age groups suggest that as people get older, they tend to watch more TV and become less active [12,16]. Such increases in sedentary behaviour and declines in physical activity are thought to risk deterioration in physical and cognitive functioning, health, and wellbeing [18-20,22,23]. Yet, few studies have described changes in TV viewing within a cohort over time. Of these, most have focused on the retirement window, finding that TV viewing time significantly increases following the transition to retirement [24]. Little evidence is available on the stability of TV viewing patterns in older adulthood in the absence of, or when controlling for, context change. Natural variation in viewing time should be accounted for when developing and estimating the effectiveness of interventions to reduce sedentary TV viewing time. Additionally, modelling the sociodemographic, behavioural and health factors associated with increased TV viewing may aid identification of subgroups of older adults who are most at risk of losses of function and health, and so in most urgent need of intervention [25]. Several observational studies have demonstrated associations between lifestyle factors and TV viewing; for example, individuals who are physically inactive, smokers, and people with obesity tend to spend more time watching TV [26]. However, it is unclear whether factors associated with TV viewing are also associated with *changes* in TV viewing.

This study drew on prospective data from a large, nationally representative cohort of UK older adults, to document changes in TV viewing duration over a two-year follow-up, and to identify socio-demographic, behavioural and health factors associated with changes in viewing time.

## Methods

### Study sample and procedures

Data were obtained from the English Longitudinal Study of Ageing (ELSA), an ongoing cohort study that contains a nationally representative sample of the English population living in households [27]. The ELSA cohort consists of men and women born on or before 29 February 1952, using multistage stratified probability sampling with postcode sectors selected at the first stage and household addresses selected at the second stage. The ELSA dataset is publicly available for research purposes, and to preserve participant anonymity, the organisation that collected data removed postcode and all other geographical identifiers from the dataset prior to release, thus precluding any analyses of potential spatial clustering effects. Participants gave full informed written consent to participate in the study and ethical approval was obtained from the London Multi-centre Research Ethics Committee.

For the purposes of the present analyses, data collected at wave 4 (2008–09, and wave 5 (2010–11) were used as these were the only waves at which TV viewing data were gathered. A total of 10,603 participants attended wave 4 (baseline), of whom 3,476 (32.8%) were excluded due to incomplete baseline data on TV viewing time, age or sex. Of the remaining 7,127 (62.7%) participants, 1,037 (14.5%) were lost to follow-up at wave 5 (follow-up), leaving a final analytic sample of 6,090 participants. Compared to those in the final sample, baseline participants subsequently excluded for any reason were older (64.9 ± 8.9 vs 65.5 ± 12.0; p = .005), more likely to be smokers (12.1% vs 17.7%; p < .001), less physically active (proportion doing no moderate or vigorous activity on a weekly basis: 17.8% vs 31.6%; p < .001), reported more depressive symptoms (11.7% vs 19.2; p < .001), of lower SES (proportion in routine or manual occupations: 36.7% vs 41.3%; p < .001), and more likely to have chronic illness (52.0% vs 56.6%; p < .001) or disability (20.9% vs 32.1%; p < .001). There were no differences in baseline TV viewing (p = .22), gender (p = .054), or obesity (p = .77).

### Measures

#### TV viewing

Data on TV viewing was collected at baseline and follow-up. Participants were asked to indicate, in whole hours, “How many hours of television do you watch on an ordinary day or evening, that is, Monday to Friday?” and “How many hours of television do you normally watch in total over the weekend, that is, Saturday and Sunday?” Average daily time spent watching TV was calculated as {((weekday TV time × 5) + (weekend TV time))/7}, so generating a mean score expressed in decimal hours. For descriptive purposes, average daily TV viewing data were classified into four categories (<2 hrs/d, 2 < 4 hrs/d, 4 < 6 hrs/d, ≥6 hrs/d). The population of the TV viewing duration categories at baseline and follow-up are described in Additional file 1: Table S1. Changes in TV viewing were expressed in minutes, following conversion of mean differences between baseline and follow-up from decimal hours to minutes.

#### Independent variables

Various demographic and health-related questions were administered by trained interviewers. Single-items were used unless otherwise specified. *Age* and *sex* were self-reported. *Retirement status* was obtained via a question about current working situation (retired, semi-retired, employed, self-employed, unemployed, permanent sick or disabled, looking after home or family, other). Participants who chose the first option were treated as retired, and all others as non-retired. *Cigarette smoking* was recorded (current, previous or non-smoker). Participants were asked how often they took part in vigorous, moderate- and low-intensity *physical activity*. Before answering, participants were shown prompt cards to help them interpret different PA intensities. Examples of moderate intensity activity included gardening, cleaning the car, walking at moderate pace, dancing, and floor or stretching exercises; vigorous intensity included running/jogging, swimming, cycling, aerobics/gym workout, tennis, and digging with a spade. Response options were: more than once a week, once a week, one to three times a month and hardly ever/never. Physical activity was further categorized into a binary variable based on reporting moderate or vigorous activity at least once a week (yes/no [28]). Self-reported *chronic illness* was recorded (yes/no). *Depressive symptoms* were assessed using the 8-item Centre of Epidemiological Studies Depression (CES-D) scale, which has been validated for use in older adults [29,30]. *Socioeconomic status (SES)* was assessed based on the last/most recent occupation and categorized into three groups (managerial/professional; intermediate; routine/manual occupations); a minority of responses that could not be reliably fitted into these categories were classified as ‘other’.

*Disability* was based on participants’ responses to questions on perceived difficulties in 6 basic activities (e.g., difficulty dressing, including putting on shoes and socks) and 7 instrumental activities of daily living (e.g., difficulty preparing a hot meal [31,32]). Participants with difficulties in one or more activities were considered to have some degree of disability.

Nurses collected anthropometric data (weight, height). Participants’ body weight was measured using Tanita electronic scales without shoes and in light clothing, and height was measured using a Stadiometer with the Frankfort plane in the horizontal position. *Body mass index* (BMI) was calculated using the standard formulae (weight [kilograms]/height [metres] squared).

### Statistical analyses

Differences in the socio-demographic, behavioural and health profile of participants in each baseline TV viewing hours category (2 hrs/d, 2 < 4 hrs/d, 4 < 6 hrs/d, ≥6 hrs/d) were examined using ANOVA for continuous and Pearson’s χ^2^ for non-continuous variables.

For illustrative purposes, the direction and magnitude of changes in viewing patterns were described for participants in each baseline TV hours category. Differences of ≥1 min in mean TV viewing duration between baseline and follow-up were treated as a change in viewing time, and differences of <1 min as no change. Paired samples t-tests were run to examine changes in average TV viewing times (continuous data) between baseline and follow-up.

Longitudinal associations between independent variables at baseline and changes in TV viewing hours between baseline and follow-up were examined using linear regression. Two incremental models were fitted: Model 1 adjusted for sex, age and baseline TV viewing, and Model 2 additionally adjusted mutually for all variables. A corresponding supplementary analysis was run to model absolute TV viewing scores at follow-up, with no adjustment for baseline TV viewing. Comparison of coefficients from this analysis, reported in Additional file 1: Table S2, with those reported in the main analysis below permits exploration of whether factors associated with TV viewing hours at follow-up retained predictive value when modeled as predictors of changes in TV viewing hours [33]. Variations of these models were also run including retirement status as a covariate, but retirement did not predict TV viewing change, or absolute TV viewing, so was not considered further. A sensitivity analysis confirmed that similar results from regression models for the full sample were found when selecting only fully retired participants (N = 3,203; 52.6% of sample); these data are reported in Additional file 1: Table S3. All analyses were conducted using SPSS v21 with statistical significance set at p < 0.05.

## Results

### Sample characteristics

Baseline sample characteristics are shown in Table 1. Participants in the highest TV viewing categories tended to have lower SES, report more depressive symptoms, smoke, be physically inactive, obese, and report chronic illness and disability.

**Table 1:** **Baseline characteristics of the sample, organised by baseline mean TV viewing time**

	***Total sample (n = 6090)***	**<2 hrs/d (n = 622)**	**2 < 4 hrs/d (n = 2077)**	**4 < 6 hrs/d (n = 1672)**	**≥ 6 hrs/d (n = 1718)**	***Test for difference***
Age (mean [SD] years)	*64.9 ± 8.9*	63.6 ± 8.9	64.4 ± 8.9	65.5 ± 9.0	65.3 ± 8.9	F = 10.12***
Men	*45.2*	53.4	47.7	42.4	41.4	χ^2^ = 37.58***
Lowest social status^‡^	*36.7*	17.8	26.2	39.8	54.0	χ^2^ = 510.22***
Depressive symptoms	*11.7*	9.5	8.1	12.1	16.4	χ^2^ = 68.55***
Disability	*20.9*	14.6	15.5	21.5	29.6	χ^2^ = 134.62***
Chronic illness	*52.0*	45.0	48.3	54.2	57.1	χ^2^ = 44.76***
Obese (BMI ≥ 30 kg/m^2^)	*31.2*	18.3	26.4	34.0	39.2	χ^2^ = 169.58***
Physically inactive^†^	*17.8*	9.0	13.3	19.9	25.0	χ^2^ = 216.26***
Current smokers	*12.1*	6.0	10.2	11.7	18.0	χ^2^ = 88.88***

Data presented are percentages unless otherwise stated.

^†^Defined as no moderate or vigorous activity on a weekly basis.

^‡^Defined as routine/manual occupations.

***p < .001.

### Observed changes in TV viewing

Weekday TV viewing time increased from 5.83 ± (SD) 5.26 hrs/d at baseline to 6.07 ± 5.37 hrs/d at follow up (p = 0.002), and weekend TV time increased from 4.05 ± 2.48 hrs/d to 4.17 ± 2.62 hrs/d at follow-up (p < 0.001). Average daily TV viewing increased from 5.32 ± 4.08 hrs/d at baseline to 5.53 ± 4.19 hrs/d at follow up (p < 0.001). The overall change in daily viewing reflected an average increase of 12 min/d although there was large variation (SD = 4.5 hrs/d). At both timepoints, participants most typically watched 2–4 hours of TV per day (baseline: N = 2,100, 34.5%; follow-up: N = 2,075, 34.2%).

While a minority of participants (N = 617; 10.1%) showed no change in TV viewing duration over the two waves (Table 2), 3,001 (49.3%) reported an increase. Of those increasing their viewing, 1,593 (53.1% of increasers; 26.2% of total sample) watched more than 60 minutes additional TV per day. Decreases in TV viewing were reported by 2,472 participants (40.6%).

**Table 2:** **Changes in mean TV viewing time between baseline and 2-year follow-up (** ***N*** **= 6,090)**

		**Change in TV viewing between baseline and follow-up** **n** ***(%)***				**TOTAL** **n** ***(%)***
		***Decrease, ≥1 min***	***No change (≥0 < 1 min difference)***	***Increase, >1 < 60mins***	***Increase, ≥60mins***	
**Baseline viewing duration** ** n ** ***(%)***	*<2 hr/d*	88 (1.4%)	110 (1.8%)	258 (4.2%)	160 (2.6%)	*616 (10.2%)*
	*2 < 4 hr/d*	567 (9.3%)	258 (4.2%)	658 (10.8%)	617 (10.1%)	*2100 (34.5%)*
	*4 < 6 hr/d*	688 (11.3%)	169 (2.8%)	361 (5.9%)	445 (7.3%)	*1663 (27.4%)*
	*≥6 hr/d*	1129 (18.5%)	80 (1.3%)	131 (2.2%)	371 (6.1%)	*1711 (28.1%)*
**TOTAL n** ***(%)***		*2472 (40.6%)*	*617 (10.1%)*	*1408 (23.1%)*	*1593 (26.2%)*	*6090 (100%)*

Percentages are of total sample.

Of those watching <2 hrs/d at baseline (N = 616), two-thirds (N = 418; 67.8%) increased their average viewing time, most commonly by 1 < 60 mins (N = 258; 41.9%), and viewing time decreased for 88 participants (14.3%). Of 2,100 participants watching 2 < 4 hrs/d at baseline, 1,275 (60.7%) increased their viewing time, most typically by 1 < 60mins (N = 658; 31.3%), and 567 (27.0%) decreased their viewing time. Around half of participants watching 4 < 6 hrs/d at baseline (N = 1,663) increased their viewing time (N = 806; 48.5%), most of whom increased by >60mins (N = 445; 26.8%), though 688 participants (41.4%) reduced viewing time. Of the 1,711 participants watching ≥6 hrs/d at baseline, 502 (29.3%) reported increases in viewing time, but the majority (N = 1,129; 66.0%) decreased their viewing time.

### Factors associated with changes in TV viewing

In models adjusting for age, sex, and baseline TV viewing, increases in TV viewing time at follow-up were associated with lower SES, depressive symptoms, disability, chronic illness, higher BMI, physical inactivity and smoking (see Table 3).

**Table 3:** **Socio-demographic, behavioural and health factors associated with changes in mean TV viewing time (in hours) between baseline and 2-year follow-up (** ***N*** **= 6,090)**

**Variable**	**N**	**Model 1 B (95% CI)**	**Model 2 B (95% CI)**
Socioeconomic status*			
Managerial/ professional	2226	Reference	Reference
Intermediate	1580	0.41 (0.16, 0.66)	0.36 (0.11, 0.60)
Manual/routine	2236	1.31 (1.08, 1.54)	1.12 (0.89, 1.36)
Other	48	-	-
*p-trend*		<0.001	<0.001
*Depression (CES-D ≥ 4)*			
No	5378	Reference	Reference
Yes	712	0.81 (0.51, 1.11)	0.43 (0.12, 0.74)
*p-trend*		<0.001	0.007
*Disability*			
No	4817	Reference	Reference
Yes	1273	0.69 (0.45, 0.94)	0.25 (−0.01, 0.52)
*p-trend*		<0.001	0.06
*Chronic illness*			
No	2924	Reference	Reference
Yes	3166	0.27 (0.08, 0.46)	−0.04 (−0.24, 0.17)
*p-trend*		0.007	0.73
*Body mass index*			
15 - 25	1631	Reference	Reference
≥25 < 30	2559	0.37 (0.13, 0.61)	0.43 (0.19, 0.66)
≥30	1900	0.91 (0.65, 1.16)	0.82 (0.56, 1.08)
*p-trend*		<0.001	<0.001
*Physical activity*			
Inactive	1085	Reference	Reference
Moderate	3017	−0.95 (−1.22, −0.68).	−0.65 (−0.92, −0.37)
Vigorous	1988	−1.07 (−1.36, −.077)	−0.57 (−0.78, −0.26)
*p-trend*		<0.001	<0.001
*Smoking*			
Never	2503	Reference	Reference
Ex-smoker	2849	0.09 (−0.12, 0.30)	0.02 (−0.19, 0.23)
Current	738	0.93 (0.61, 1.25)	0.71 (0.39, 1.03)
*p-trend*		<0.001	<0.001

**Model 1** adjusted for age, sex and baseline TV viewing. **Model 2** adjusted for age, sex, baseline TV viewing and mutually for all variables presented. Β (95% Confidence Interval) coefficients reflect increases in hours/day of TV viewing between baseline and follow-up. * Coefficients are not reported for the ‘other’ socioeconomic status category due to small sample size and heterogeneity of employment statuses captured by this category. All available data were however entered into the regression model, in that coefficients for other socioeconomic status categories were calculated using dummy variables that contrasted the focal category with all three other categories.

When also mutually controlling for all variables as covariates, associations for SES, depression, BMI, physical activity, and smoking status remained. Hours of TV viewing increased more markedly among participants in intermediate (B = 0.36 [95% CI: 0.11, 0.60]) or manual/routine social occupational classes (B = 1.12 [0.89, 1.36]; p < .001) compared to those of managerial/professional status. Participants with depressive symptoms increased their TV viewing more than did those without (B = 0.43 [0.12, 0.74], p = .007). TV viewing increased more among overweight or obese participants (BMI ≥25 < 30 B = 0.43 [0.19, 0.66]; BMI ≥30 B = 0.82 [0.56, 1.08]; p < .001) than among those of normal weight or underweight, and decreased over time in physically active participants (relative to inactivity, moderate B = −0.65 [−0.92, −0.37]; vigorous B = −0.57 [−0.78, −0.26]; p < .001). Relative to never-smokers, current smokers reported greater increases in TV viewing (B = 0.71 [0.39, 1.03]; p < .001), but no association was found among ex-smokers (B = 0.02 [−0.19, 0.23]). There were no associations of disability (p = .06) or chronic illness (p = .73) with changes in TV viewing.

## Discussion

This prospective study of a large and nationally representative cohort of older adults in England sought to describe changes in participants’ TV viewing time over a 2-year follow-up period, and model socio-demographic, behavioural and health factors associated with increases in TV viewing. Results showed that participants watched an average of over 5 hours of TV at baseline, and mean viewing time across the whole cohort increased slightly but significantly over time. While 41% of participants decreased their viewing over the two waves, half of participants increased their viewing, with a quarter of the sample watching at least one more hour of TV daily at follow-up. Increases in TV viewing were associated with lower socioeconomic status, depressive symptoms, higher BMI, lower levels of physical activity, and being a smoker.

To our knowledge, our study is one of the first to use prospective data to describe variation in TV viewing over time. In addition to observing mean increases in viewing time, we also investigated patterns of change according to baseline viewing duration. These showed a general tendency towards increased viewing among participants viewing fewer than 6 hours of TV per day at baseline. While the majority of those watching 6 or more hours per day at baseline reported decreased viewing time at follow-up, 29% of this group increased their viewing time by at least one hour. Previous research has suggested that the transition to retirement is associated with increases in TV viewing [24], but we found that retirement status did not predict change in viewing duration. Additionally, similar patterns of results were observed among a subsample of participants who were fully retired at baseline, though depression and smoking predicted viewing change among the full sample but not among the retired. Our findings thus testify to the potential for, and magnitude of, naturally occurring increases in TV viewing among older adults over time, even where accounting for the retirement transition.

Our data support previous studies by demonstrating associations between TV viewing and behavioural and psychosocial variables [26]. For example, a large Belgian cross-sectional sample found higher levels of TV viewing among functionally limited, less educated, widowed, and (semi-)urban dwelling older adults [34]. In a community sample of older Japanese adults, TV time was associated with not being in full-time employment, lower educational attainment, increased weight, living in regional areas and low moderate-to-vigorous physical activity [35]. Yet, factors associated with variation in static TV viewing scores need not be associated with changes in TV viewing over time; indeed, though our supplementary analysis showed that most predictors of TV viewing time also predicted changes in viewing time, chronic illness was associated with greater TV viewing time at follow-up but not increases in viewing time between waves. Our results showed that older adults with lower SES, depression, overweight and obese, physical inactivity, and smokers were more likely to increase their TV viewing. These subgroups may therefore require especial attention, with a particular focus on cardiovascular and respiratory disorders and mental health problems, when developing sedentary behaviour reduction interventions.

Our findings call for the development of effective behaviour change interventions to reduce sedentary behaviour among older adults. That more physically active participants tended to decrease their TV viewing time over the two waves concurs with previous research showing that engagement in physical activity can reduce time spent in sedentary activity [12,36]. Sedentary behaviour change interventions might most usefully seek to displace TV viewing minutes with physical activities. Calls have been made for interventions to encourage older adults to get ‘out and about’ as a means of displacing sedentary home-based activities such as TV viewing [37]. Yet, seasonal changes, and a perceived lack of safety and security in the local neighbourhood, can limit the effectiveness of non-home-based interventions among the elderly [38]. TV viewing is often driven by enjoyment of TV [39], which may constrain the acceptability of interventions to replace TV viewing with alternative, non-sedentary activities. Given growing evidence of the positive health impacts of minimal-intensity physical activity relative to sedentary behaviour [40-42], it may be feasible and beneficial to health to incorporate light-intensity physical activities into TV viewing patterns. Several potentially low-intensity activities have been proposed for insertion into otherwise sedentary TV viewing periods so as to promote physical activity and reduce sitting time, such as marching on the spot during commercial breaks [43], doing chores [44], operating a foot pedal device [45], or merely standing up, as a balance activity [46]. Frequent performance of such activities has the potential to maintain the muscle power, balance and confidence required to stay physically active in older adulthood [47]. Additionally, behavioural psychology suggests that adding activity into stable TV viewing routines would be particularly conducive to the formation of physical activity habits: consistent repetition of physical activities while watching TV should lead, through associative learning, to the activities becoming automatically activated with minimal mental effort when watching TV [48,49]. In this way, physical activity should become an ingrained part of the TV viewing routine [49].

Limitations of this study should be acknowledged. First, the self-report TV viewing measure used has not been validated. However, many objective accelerometry devices cannot reliably differentiate sitting from other forms of light ambulatory movement, precluding true validation of self-reported sedentary behaviour measures. In the absence of such data, it is notable that the TV measure used in the present dataset has demonstrated convergent validity with various psychosocial, physical and biochemical risk factors hypothesised to be linked with sedentary behaviour [18,19,50]. Second, given the paucity of data on changes in TV viewing duration over time, it is unclear to what extent TV viewing patterns that we have reported are likely to replicate to other samples and settings. Participants reported watching TV for an average of 4.7 hours per day at baseline and 4.9 at follow-up. This exceeds the average 4 daily hours of TV time observed among older adults in a 2005 UK survey [16], and the 3.0-3.5 daily hours observed in UK EPIC-Norfolk data [51]. Levels of sedentary behaviour may differ considerably both across and within nations [11], making it difficult to estimate the generalisability of our data. Relatedly, our analytic sample was healthier and more active than those who were lost to follow-up. Inactive people must be engaged in research for findings to have external validity. Third, all measures were self-reported, and so actual TV viewing time may have been underestimated. In addition, participants may have reported the amount of time the TV was turned on but not necessarily for how long they watched it. Population-based data should ideally employ both self-report and objective measures for verification purposes [52]. It is also unclear whether TV viewing self-reports were consistently accurate over time. Some of the observed variation in TV viewing duration may be attributable to a lack of measurement stability, and it is not possible to isolate true changes in TV time from method error due to inconsistent reporting. More data is needed to evaluate the reliability, replicability and generalisability of our findings to other samples. Fourth, we focused on TV viewing duration, but not the times at which TV viewing occurred. It is unclear whether TV viewing minutes were mostly accrued in prolonged bouts of sitting, or are dispersed over multiple shorter viewing periods throughout the day. Complementing reports of total TV time with accelerometry, so as to obtain time-stamped data on when TV viewing most typically occurs, could aid the development of time-appropriate sedentary reduction intervention strategies [12].

## Conclusions

Half of all participants in this large cohort study of older adults increased the time they spent watching TV over a two-year time period. These results are important in documenting natural variation in TV viewing time within a cohort over time, in the absence of intervention. Individuals with lower SES, depressive symptoms, higher BMI, physical inactivity, and smokers exhibited greater increases in TV viewing time. These subgroups represent priority targets for interventions to reduce sedentary behaviour among older adults.

## Additional file

Additional file 1: Table S1. Changes in TV viewing duration categories between baseline and 2-year follow-up (*n* = 6,090). **Table S2**. Socio-demographic, behavioural and health factors associated with TV viewing time (in hours) at 2-year follow-up (no adjustment for baseline TV viewing; *n* = 6,090).** Table S3**. Socio-demographic, behavioural and health factors associated with changes in TV viewing time (in hours) between baseline and 2-year follow-up in retired participants (*n* = 3,203).

## Abbreviations

BMI: Body mass index
CI: Confidence interval
CES-D: Centre of Epidemiological Studies Depression scale
ELSA: English Longitudinal Study of Ageing
SD: Standard deviation
SES: Socioeconomic status
TV: Television
UK: United Kingdom
