# Supplementary material for: Sociodemographic, behavioural and health factors associated with changes in older adults’ TV viewing over 2 years
Source: Int J Behav Nutr Phys Act. 2014 Aug 15;11:102. doi: 10.1186/s12966-014-0102-3 (PMC4149242; doi:10.1186/s12966-014-0102-3)
Supplement: Additional file 1: Table S1. — Changes in TV viewing duration categories between baseline and 2-year follow-up (n = 6,090). Table S2. Socio-demographic, behavioural and health factors associated with TV viewing time (in hours) at 2-year follow-up (no adjustment for baseline TV viewing; n = 6,090). Table S3. Socio-demographic, behavioural and health factors associated with changes in TV viewing time (in hours) between baseline and 2-year follow-up in retired participants (n = 3,203). [file 12966_2014_102_MOESM1_ESM.docx]

**Supplementary Table 1.** Changes in TV viewing duration categories between baseline and 2-year follow-up (*n* = 6,090)

|  | | **Follow-up**  n *(%)* | | | | TOTAL  n *(%)* |
| --- | --- | --- | --- | --- | --- | --- |
| **Baseline**  n *(%)* |  | *<2hr/d* | *2<4hr/d* | *4<6hr/d* | *≥6hr/d* |  |
|  | *<2hr/d* | 320  (5.3%) | 223  (3.7%) | 28  (0.5%) | 45  (0.7%) | *616*  *(10.2%)* |
|  | *2<4hr/d* | 141  (2.3%) | 1209  (19.9%) | 433  (7.1%) | 317  (5.2%) | *2100*  *(34.5%)* |
|  | *4<6hr/d* | 22  (0.4%) | 363  (6.0%) | 805  (13.2%) | 473  (7.8%) | *1663*  *(27.4%)* |
|  | *≥6hr/d* | 21  (0.3%) | 280  (4.6%) | 435  (7.1%) | 975  (16.0%) | *1711*  *(28.1%)* |
| TOTAL  n *(%)* |  | *504*  *(8.3%)* | *2075*  *(34.2%)* | *1701*  *(27.9%)* | *1810*  *(29.7%)* | *6090*  *(100%)* |

NB: Percentages are of total sample.

**Supplementary Table 2.** Socio-demographic, behavioural and health factors associated with TV viewing time (in hours) at 2-year follow-up (no adjustment for baseline TV viewing; *n* = 6,090).

| **Variable** | **N** | **Model 1**  **B (95% CI)** | **Model 2**  **B (95% CI)** |
| --- | --- | --- | --- |
| *Socioeconomic status* |  |  |  |
| Managerial/ professional | 2226 | Reference | Reference |
| Intermediate | 1580 | 0.73 (0.47, 1.00) | 0.63 (0.36, 0.89) |
| Manual/routine | 2236 | 2.18 (1.94, 2.42) | 1.83 (1.58, 2.00) |
| *p-trend* |  | <0.001 | <0.001 |
| *Depression (CES-D≥4)* |  |  |  |
| No | 5378 | Reference | Reference |
| Yes | 712 | 1.24 (0.91, 1.57) | 0.58 (0.26, 0.91) |
| *p-trend* |  | <0.001 | <0.001 |
| *Disability* |  |  |  |
| No | 4817 | Reference | Reference |
| Yes | 1273 | 1.12 (0.86, 1.39) | 0.37 (0.09, 0.65) |
| *p-trend* |  | <0.001 | 0.01 |
| *Chronic illness* |  |  |  |
| No | 2924 | Reference | Reference |
| Yes | 3166 | 0.48 (0.27, 0.69) | -0.02 (-0.23, 0.20) |
| *p-trend* |  | <0.001 | 0.89 |
| *Body mass index* |  |  |  |
| 15 - 25 | 1631 | Reference | Reference |
| ≥25<30 | 2559 | 0.58 (0.32, 0.84) | 0.66 (0.36, 0.87) |
| ≥30 | 1900 | 1.43 (1.16, 1.71) | 1.18 (0.91, 1.45) |
| *p-trend* |  | <0.001 | <0.001 |
| *Physical activity* |  |  |  |
| Inactive | 1085 | Reference | Reference |
| Moderate | 3017 | -1.27 (-1.64, -0.98) | -0.74 (-1.03, -0.45) |
| Vigorous | 1988 | -1.70 (-2.00, -1.78) | -0.83 (-1.15, -0.50) |
| *p-trend* |  | <0.001 | <0.001 |
| *Smoking* |  |  |  |
| Never | 2503 | Reference | Reference |
| Ex-smoker | 2849 | 0.22 (-0.004, 0.25) | 0.10 (-0.12, 0.31) |
| Current | 738 | 1.47 (1.13, 1.81) | 1.00 (0.65, 1.33) |
| *p-trend* |  | <0.001 | <0.001 |

**Model 1** adjusted for age and sex. **Model 2** adjusted for age, sex, and mutually for all variables presented. B (95% Confidence Interval) coefficients reflect hours/day of TV viewing.

**Supplementary Table 3.** Socio-demographic, behavioural and health factors associated with changes in TV viewing time (in hours) between baseline and 2-year follow-up in retired participants (*n* = 3,203).

| **Variable** | **N** | **Fully adjusted model**  **B (95% CI)** |
| --- | --- | --- |
| *Socioeconomic status* |  |  |
| Managerial/ professional | 1179 | Reference |
| Intermediate | 815 | 0.28 (-0.06, 0.63) |
| Manual/routine | 1178 | 1.17 (0.86, 1.79) |
| *p-trend* |  | <0.001 |
| *Depression (CES-D≥4)* |  |  |
| No | 2831 | Reference |
| Yes | 372 | 0.32 (-0.10, 0.73) |
| *p-trend* |  | 0.139 |
| *Disability* |  |  |
| No | 2370 | Reference |
| Yes | 833 | 0.30 (-0.04, 0.63) |
| *p-trend* |  | 0.078 |
| *Chronic illness* |  |  |
| No | 1360 | Reference |
| Yes | 1843 | 0.19 (-0.09, 0.47) |
| *p-trend* |  | 0.18 |
| *Body mass index* |  |  |
| 15 - 25 | 815 | Reference |
| ≥25<30 | 1435 | 0.40 (0.08, 0.73) |
| ≥30 | 953 | 0.75 (0.39, 1.11) |
| *p-trend* |  | <0.001 |
| *Physical activity* |  |  |
| Inactive | 661 | Reference |
| Moderate | 1590 | -0.65 (-1.01, -0.29) |
| Vigorous | 952 | -0.61 (-1.02, -0.19) |
| *p-trend* |  | 0.001 |
| *Smoking* |  |  |
| Never | 1282 | Reference |
| Ex-smoker | 1629 | 0.14 (-0.14, 0.42) |
| Current | 292 | 0.59 (0.10, 1.08) |
| *p-trend* |  | 0.062 |

Model adjusts for age, sex, baseline TV viewing, and mutually for all variables presented. B (95% Confidence Interval) coefficients reflect increases in hours/day of TV viewing between baseline and follow-up. Retired participants were those self-classifying as ‘fully retired’ at baseline.
